# Small RNA Expression from the Human Macrosatellite DXZ4

**DOI:** 10.1534/g3.114.012260

**Published:** 2014-08-21

**Authors:** Michael Pohlers, J. Mauro Calabrese, Terry Magnuson

**Affiliations:** * Department of Genetics, the Carolina Center for Genome Sciences, University of North Carolina, Chapel Hill, North Carolina 27599; † Lineberger Comprehensive Cancer Center, University of North Carolina, Chapel Hill, North Carolina 27599

**Keywords:** small noncoding RNA, somatic cells, DNA methylation, Argonaute, PIWI

## Abstract

Small noncoding RNAs play several roles in regulating gene expression. In the nucleus, small RNA-Argonaute complexes recruit epigenetic modifying activities to genomic sites. This pathway has been described in mammals primarily for the germline; however, its role in somatic cells is less characterized. Here, we describe in human somatic cells a potential link between the expression of small RNAs from the macrosatellite *DXZ4* and Argonaute-dependent DNA methylation of this locus. *DXZ4* was found to express a wide range of small RNAs potentially representing several classes of small RNAs. A subpopulation of these RNAs is bound by Argonaute. Moreover, we show AGO association with *DXZ4* and that the Argonaute proteins AGO-1 and PIWIL4 may play a role in DNA methylation of *DXZ4*. We hypothesize that the RNAs are involved in Argonaute-dependent methylation of *DXZ4* DNA.

At least half of the human genome is characterized as repetitive sequence that does not code for proteins. One of the two main families of DNA repeats are tandem repeats, including satellite DNA. *DXZ4* is one of several macrosatellites identified in humans and consists of 50−100 copies of a 3-kb monomer (Giacalone *et al.* 1992). By virtue of its location on the X chromosome, *DXZ4* is subject to X chromosome inactivation. However, contrary to what is expected, *DXZ4* on the inactive X chromosome is highlighted by a euchromatic conformation consisting of dimethylated histone H3 lysine-4 (H3K4me2) and acetylated H3 lysine-9 (H3K9Ac) (Boggs *et al.* 2002; Chadwick 2008). In contrast, on the active X chromosome in both female and male cells, *DXZ4* chromatin contains the heterochromatin-associated H3K9me3 modification (Chadwick 2008). Moreover on the active X, *DXZ4* DNA is hypermethylated, also a characteristic of the repressed transcription state, and in contrast, hypomethylated on the inactive X (Giacalone *et al.* 1992; Chadwick 2008).

Studies in the mouse germline have demonstrated that Piwi-interacting RNAs (piRNAs) re-establish *de novo* DNA methylation in males (Carmell *et al.* 2007; Aravin *et al.* 2008; Kuramochi-Miyagawa *et al.* 2008). This methylation is important for restricting transposon mobility. It has also been demonstrated that piRNA-mediated targeting induces allele-specific *Rasgrf1* transcriptional silencing by *de novo* DNA methylation, thereby resulting in germline genomic imprinting of this locus (Watanabe *et al.* 2011). Recently, piRNA-like molecules have been identified in somatic tissues from multiple organisms (Ro *et al.* 2007; Ghildiyal *et al.* 2008; Li *et al.* 2009; Yan *et al.* 2011). A particularly convincing example is the induction of piRNAs in the central nervous system of the sea slug *Aplysia* by the neurotransmitter serotonin (Rajasethupathy *et al.* 2012). In neurons, a specific piRNA is induced by serotonin and inhibition of the Piwi protein abolished the serotonin-dependent methylation increase of a CpG island within the promoter of the *CREB2* gene whose protein product is important for the persistence of memory. In human tissue culture cells, the PIWI protein PIWIL4 can induce histone H3 lysine 9 dimethylation (H3K9me2) at the *p16^Ink4a^* locus, resulting in down-regulation of the gene (Sugimoto *et al.* 2007). Also in tissue culture cells, microRNAs (miRNAs) have been found to interact with promoters to induce transcriptional gene silencing (Huang and Li 2012). Furthermore, endogenous small interfering RNAs (siRNAs) have been shown to be involved in H3K9me2 deposition at several genomic repeats to regulate genome stability in fruit fly somatic cells (Peng and Karpen 2007; Fagegaltier *et al.* 2009). Taken together, this evidence suggests that small RNA pathways can regulate gene expression by chromatin modification in somatic cells.

Because small RNAs are known to act on repetitive sequences (Peng and Karpen 2007; Fagegaltier *et al.* 2009), together with the fact that *DXZ4* expresses ~85 nucleotide long RNAs (Chadwick 2008), we investigated whether small RNAs are expressed from *DXZ4*. In somatic cells, we detected the expression of a wide range of small RNAs from this locus with a subpopulation associating with Argonaute. Based on this finding, we further investigated whether the small RNA pathway plays a role in establishing epigenetic modifications at *DXZ4* and found that Argonaute proteins are required for DNA methylation and that they bind *DXZ4* chromatin. We speculate that the small RNAs are involved in the establishment of epigenetic modifications at this region.

## Materials and Methods

### Small RNA Northern hybridization

A total of 30 μg of total RNA was isolated using TriZol (Life Technologies). The RNA population <200 nucleotides was isolated from 5.5 × 10^6^ cells (PureLink miRNA isolation kit; Life Technologies). Extracting the chromatin fraction from 5.5 × 10^6^ cells was published elsewhere (Kugler *et al.* 1995). However, total RNA from the chromatin fraction was isolated using TriZol. RNA was separated on a denaturing 15% polyacrylamide gel, transferred to a HyBond-N membrane (GE Healthcare), ultraviolet crosslinked, and probed with 5′ ^32^P-phosphorylated oligonucleotides in ExpressHyb solution (Clontech) at 37°. Probes: DXZ4 1536-bp tgacgactcgtgtgtgccgtgg, DXZ4 2352-bp acacctatccccctggctcg, DXZ4 2942-bp ccccgggcccccttagccgatg. Probes for 3′ probing: DXZ4 cgcccccacgggaccgctctcgagg, cacacctatccccctggctcgctct, gcgagagcggtccgccgtgcccaag; miR-15a cacaaaccattatgtgctgcta.

### Small RNA detection by quantitative reverse-transcription polymerase chain reaction (RT-PCR)

Custom TaqMan small RNA assays (Life Technologies) were performed according to the manufacture’s protocol using 500 ng of RNA <200 nucleotides. A U6 snoRNA probe (Life Technologies) was used as internal standard. Probed small RNAs included the following: DXZ4-2183as tcaccttggcttgggggacctcgagagcggtcccgt, DXZ4-2259as cgtcaacgcacctttaagggcgagagcggtccgccg, and DXZ4-2355as cctatccccctggctcgctctc.

### RNA interference

We transiently transfected 8 × 10^4^–3.5 × 10^5^ HEK293T cells or 2 × 10^5^ MRC-5 cells per 12-well dish with 5–20 nM siRNA the following day after seeding using Lipofectamine RNAiMax (Life Technologies). Cells were analyzed 48–72 hr posttransfection. Silencer Select Validated siRNAs (Life Technologies) were as follows: negative control no. 1 4390843; AGO-1 s25500, s25501; PIWIL4 s44573; Dicer s23756.

### *In vitro* Drosha cleavage assay

*In vitro* Drosha processing was performed essentially as described (Zeng and Cullen 2006), except that nuclear extracts were prepared from HEK293T cells transfected with expression plasmids for Drosha and DGCR8 and used directly in the assay instead of using purified recombinant proteins.

### Probing of 3′ RNA ends

NaIO_4_ treatment and β-elimination was performed as described (Vagin *et al.* 2006).

### Chromatin immunoprecipitation (ChIP)

ChIP was performed essentially as described with minor adaptations (Kim and Rossi 2009). A total of 5 µg of anti-pan-AGO (Z. Mourelatos and EMD Millipore MABE56) was preconjugated to 25 µL of protein A/G Plus-agarose-beads (sc-2003; Santa Cruz Biotechnology) in 0.5% bovine serum albumin/phosphate-buffered saline (PBS) for at least 6 hr. Trypsinized HEK293T cells were crosslinked with 1% formaldehyde for 10 min at room temperature in Dulbecco’s Modified Eagle Medium and 10% serum, followed by a 5-min quench with 125 mM glycine. Cell pellet was resuspended in ChIP lysis buffer (50 mM HEPES at pH 7.5, 140 mM NaCl, 10% Triton X-100, 0.1% sodium deoxycholate, protease inhibitors) and incubated on ice. After 10 min, samples were centrifuged, pellets resuspended again in ChIP lysis buffer, and incubated for 10 min on ice. Extracts were then sonicated to generate ~200- to 500-bp DNA fragments and cleared via centrifugation for 10 min. Then, 300 µg of chromatin was added to the antibody-coated beads and incubated overnight. Beads were washed at 4° 1× with 1 mL of ChIP lysis buffer, 2× with 1 mL of ChIP lysis buffer high salt (50 mM HEPES at pH 7.5, 500 mM NaCl, 1% Triton X-100, 0.1% sodium deoxycholate), followed by two washes with 1 mL of ChIP wash buffer (10 mM Tris-HCl at pH 8.0, 250 mM LiCl, 0.5% NP-40, 0.5% sodium deoxycholate, 1 mM EDTA). Antibody complexes were eluted 2× for 10 min at 65° in (50 mM Tris pH 8.0, 10mM EDTA, and 1% sodium dodecyl sulfate), crosslinks were reversed at 65° for 4 hr. Eluates were incubated with Proteinase K, and DNA was extracted with phenol/chloroform and ethanol-precipitated.

#### Semiquantitative detection of PIWIL4 mRNA expression:

For RT-PCR, 1 µg of total RNA was reverse transcribed using oligo d(T) primers. PIWIL4 cDNA was amplified for 30 cycles with the following primer pair: PIWIL4s CCTGATGGTGGTCGGTATTGA and PIWIL4as ACACAATTATCCGTGCTGGC.

### DNA methylation analysis

Part of the siRNA-transfected cell population was used to assess knockdown efficiency by quantitative RT-PCR. Genomic DNA from the remaining cells was isolated and bisulfite modified with the EpiTect Plus DNA Bisulfite kit (QIAGEN). Modified DNA was amplified, subcloned, and sequenced. *DXZ4* and *H19* primers specific to bisulfite-modified DNA have been published elsewhere (Borghol *et al.* 2006; Chadwick 2008). Sequence reads were analyzed with the QUMA online tool (Kumaki *et al.* 2008).

### Fluorescence *in situ* hybridization (FISH)

For the *DXZ4* FISH probe, a full-length *DXZ4* monomer was PCR-amplified from human fibroblast genomic DNA (5′-gctttgccaccgaactcatcg, 5′-aagcttgagaaatggagactc) and gel eluted. For the XIST FISH probe, a bacterial artificial chromosome plasmid (RP11-13M9, CHORI BPRC) was used. Then, 25 ng of probe DNA was labeled with the BioPrime DNA labeling kit (Life Technologies), using Cy3- or Cy5-conjugated dCTP (GE Healthcare), and stored in 70% ethanol at −20° until further use. To prepare FISH probes for hybridization, probes were precipitated with yeast transfer RNA (tRNA) and Salmon Sperm DNA (both Life Technologies). After washes with 75% and 100% ethanol, probes were air-dried and denatured for 10 min in 50–100 µL of 100% formamide at 85°. An equal volume of 2× hybridization buffer (25% dextran sulfate/4× saline sodium citrate [SSC]) was then added, and probes were prehybridized for 90 min at 37°.

DNA and RNA FISH experiments were performed essentially as described (Calabrese *et al.* 2012). Cells were grown on coverslips, fixed for 10 min in 2% paraformaldehyde/PBS at room temperature, and permeabilized for 10 min on ice in 0.5% Triton X-100/PBS. Cells were then dehydrated by serial 3-min incubations with 75%, 85%, 95%, and 100% ethanol and air-dried for 5 min. For combined DNA/RNA FISH experiments, cells were heat denatured at 80° for 9 min in 70% formamide/2× SSC followed by two washes in cold 2× SSC. Probes were jointly hybridized overnight at 37°. Coverslips were washed 2× for 5 min in 50% formamide/2× SSC at 37°, then 2× for 5 min in 2× SSC at 37°. DAPI was added at 100 ng/mL to one of the wash buffers.

## Results and Discussion

### *DXZ4* expresses a wide range of chromatin-associated small RNAs

**Figure 1:**
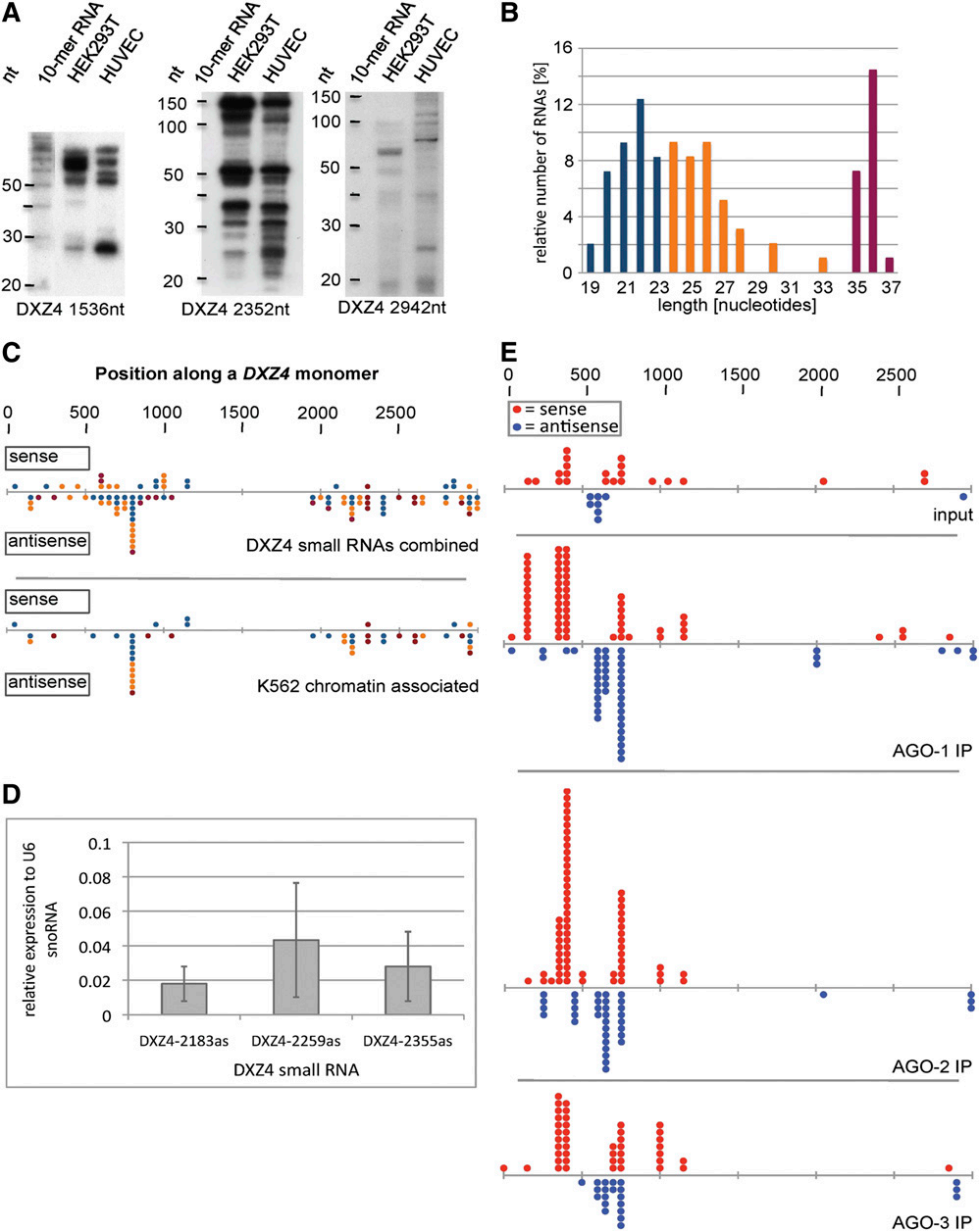
Small RNA expression from *DXZ4*. (A) Northern hybridization detecting small RNAs expressed from *DXZ4* in the cell lines HEK293T and human umbilical vein endothelial cell (HUVEC). The numbers below correlate with the start position of the Northern probes in *DXZ4*, which colocalize with *DXZ4* regions associated with characteristic histone modifications. (B) Distribution of the lengths of *DXZ4*-matching unique small RNA reads obtained from merged high-throughput sequencing data from K562, HEK293, MCF10a, H1, and IMR-90 cell lines (n = 97). Coloring corresponds to size range of known small RNA classes. (C) Alignment of *DXZ4* small RNAs from (B) and from the K562 chromatin fraction only (n = 47), respectively. Refer to the text for details. The colors of the dots reflect the colors used in (B) indicating small RNA size. (D) Relative expression of *DXZ4* small RNAs in HEK293T detected by quantitative reverse-transcription polymerase chain reaction. cDNA synthesis was performed in the presence or absence of reverse transcriptase. Each reaction was performed n ≥ 6. (E) Alignment of sequenced *DXZ4* small RNAs coprecipitated in AGO immunoprecipitations in HeLa cells. Alignment includes multiple RNA copies.

Northern blots with RNA from the cell lines HEK293T and human umbilical vein endothelial cell were probed for regions that express 85-nucleotide *DXZ4* RNAs detecting multiple transcripts including small RNAs between 20 and 40 nucleotides long (Figure 1A). This size range corresponds to several classes of small RNAs known to mediate epigenetic modifications (Kim *et al.* 2009). In addition to these small RNAs, the probes detected several longer transcripts. Because of their design, the probes are predicted to detect the 85-nucleotide transcripts as well as any larger transcripts (Chadwick 2008), providing a putative explanation for the detection of multiple signals per probe. However, although we used stringent hybridization and washing conditions, we cannot exclude the presence of unintended cross hybridization signals.

Further, we identified *DXZ4*-matching small RNAs from several published high-throughput small RNA sequencing studies from a total of 3.2 × 10^7^ reads (Fejes-Toth *et al.* 2009; Mayr and Bartel 2009; Bernstein *et al.* 2010). Up to three mismatches were tolerated to factor in single-nucleotide polymorphisms between *DXZ4* monomers, potential posttranscriptional editing, and sequencing errors (Tremblay *et al.* 2011). More specifically, we considered only reads that did not coalign to other genomic sites with the same or a greater stringency than to *DXZ4* (Figure 1, B and C). This filtration led to the exclusion, among others, of all small RNAs aligning to a continuous stretch of 775 nucleotides in the center of the *DXZ4* monomer representing 38% of highly stringent *DXZ4* reads. The region includes 664 nucleotides with more than 99% identity to an intronic sequence of *ARID5b* and is followed by a CT repeat. A total of 55% of the remaining 97 unique *DXZ4* small RNA sequences matched perfectly. The sequence data confirmed the specificity of our small RNA Northern, because many of the probes we used were identical or partially overlapped with sequenced small RNAs, for example the probes employed generating the data in Figure 1A. The relative small number of identified unique reads indicates a low expression level *DXZ4* small RNAs. Approximately 25% of the small RNA sequences were in the sense direction and 75% in the antisense direction. Interestingly, almost 22% of the sequences were 35, 36, or 37 nucleotides long (Figure 1B). Although piRNAs do not typically exceed 32 nucleotides, those with 36 nucleotides have been found (Ro *et al.* 2007). Alternatively, the *DXZ4* complementary sequences could be derived from other classes of small RNAs, for example, tRNA-derived small RNAs, which can reach a length of 38 nucleotides (Lee *et al.* 2009; Peng *et al.* 2012). However, the *DXZ4* sequence did not reveal any annotated transfer-RNA genes, nor could we identify tRNA-like sequences using tRNA search programs (Laslett and Canback 2004; Schattner *et al.* 2005).

Because *DXZ4* expresses 85-nucleotide RNAs from regions characterized by specific histone modifications (Chadwick 2008), we plotted the position and size of the *DXZ4* small RNAs identified by high-throughput sequencing relative to the *DXZ4* monomer (Figure 1C). Sequenced RNAs associated with chromatin also were analyzed individually to reveal any potential link between the region of specific histone modifications and the genomic origin of the small RNAs. In general, the results show that the *DXZ4* small RNA expression was not restricted to regions previously shown to harbor specific histone modifications. However, a number of *DXZ4* sites, in particular around nucleotide 800, were associated with an increased number of small RNAs. This enrichment was mostly not generated by multiple copies of the same sequences but instead by slightly different RNAs. We did not recognize a correlation between the lengths of the small RNAs and their origins (Figure 1C).

To validate the expression of *DXZ4* small RNAs via an alternate method, we performed quantitative RT-PCR with RNA from HEK293T, MRC-5, and IMR-90 cells. We chose RNA species that were identified in the high-throughput sequencing datasets. The detection of expression of all three small RNAs in HEK293T cells validated the existence of *DXZ4* small RNAs by an additional independent method (Figure 1D). The late amplification signals corroborate a low expression level of these small RNAs as indicated in the sequencing data analysis earlier. One of the small RNA assays also detected expression in MRC-5 cells. However, the small RNAs were barely or not detectable in IMR-90, respectively. This differential small RNA expression correlates with our observations identifying these RNAs by Northern hybridization and in the sequencing datasets in some cell lines but not in all.

The presence of *DXZ4* small RNAs in the K562 chromatin fraction prompted us to investigate chromatin-associated RNAs in MRC-5, IMR-90, and HEK293T cells. We explored their presence in a 300-base pair *DXZ4* window by a Northern hybridization-based scanning approach using consecutive oligonucleotide probes. This sequence window encompasses one of the known regions containing histone modifications (Chadwick 2008). Many small RNAs in both orientations were detected, including some that were specific to one or two of the cell lines corroborating results described previously (Supporting Information, Figure S1A and B). In agreement with the sequencing data, small RNAs detected by Northern hybridization from the chromatin fraction originated from sites dispersed along the entire monomer sequence.

Association of *DXZ4* small RNAs with Argonaute proteins would strongly support a function of these small RNAs. We therefore searched for *DXZ4* small RNAs in co-precipitated RNA populations from Argonaute immunoprecipitations (Dueck *et al.* 2012). Matching sequences were filtered by the same parameters as specified previously. AGO-1, AGO-2, and AGO-3 are expressed in HeLa cells and were associated with *DXZ4* small RNAs in both orientations (Figure 1E). A total of 55% of the incorporated reads matched perfectly to *DXZ4*. In contrast, the IgG control and the coprecipitated material from AGO-4, which is not expressed in these cells, did not reveal any *DXZ4* sequences. Aligning the identified sequences to the *DXZ4* monomer showed a concentration in the 5′ region that includes but also extends beyond the promoter (Figure 1E).

### Characterization of *DXZ4* small RNAs

**Figure 2:**
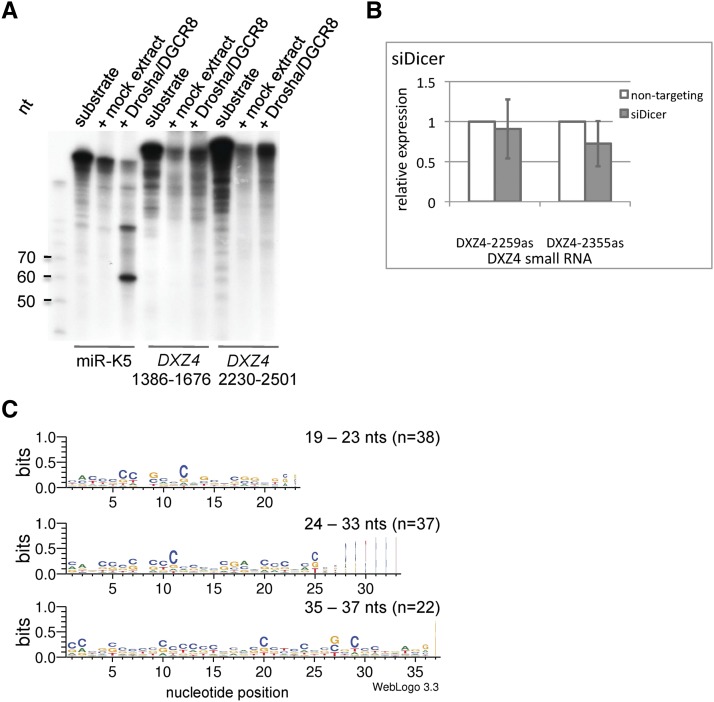
Characterization of *DXZ4* small RNAs. (A) *In vitro* Drosha assay with extracts from HEK293T cells either mock transfected or cotransfected with expression plasmids for Drosha and DGCR8. Radiolabeled *in vitro* transcripts from two *DXZ4* regions characterized by specific histone modifications and the KSHV precursor-miRNA miR-K5 served as substrates for Drosha/DGCR8 processing and were separated by polyacrylamide gel electrophoresis. (B) Effect of Dicer depletion on *DXZ4* small RNA expression in HEK293T cells. Relative expression of two small RNAs was determined by quantitative reverse-transcription polymerase chain reaction (n = 5). The difference with DXZ4-2355as is not statistically significant (P-value = 0.089, *t*-test). (C) Sequence logos for *DXZ4* small RNAs grouped according to their lengths.

The size range of *DXZ4* small RNAs corresponds to several classes of small RNAs, including siRNAs, miRNAs, and piRNAs (Kim *et al.* 2009). To characterize these RNAs, we tested processing of potential precursor-miRNAs by the Drosha/DGCR8 complex (Lee *et al.* 2003; Denli *et al.* 2004; Gregory *et al.* 2004). Because processing requires folding of the primary miRNA into a characteristic hairpin stem loop structure (Zeng *et al.* 2005), approximately 65-nucleotide long *DXZ4* RNA fragments were tested with a secondary structure algorithm (Zuker 2003). For several fragments, one or several thermodynamically stable hairpin structures were identified, implying *DXZ4* transcripts could resemble precursor-miRNAs. Two of those potential precursors were analyzed in a Drosha cleavage assay (Zeng and Cullen 2006). The Kaposi’s sarcoma-associated herpesvirus miR-K5 precursor that previously had been identified as a substrate (Gottwein *et al.* 2006) yielded the expected ~65 nucleotide cleavage product (Figure 2A). In contrast, both *DXZ4* transcripts were not cleaved, suggesting they are not proper Drosha substrates. It has been shown that subtle differences in the stem-loop structure can severely impair its ability to be processed (Gottwein *et al.* 2006). Although those *DXZ4* fragments could potentially fold into stem loops, we conclude that the limited number of *DXZ4* RNA fragments tested is not regular precursor-miRNAs. It is known that some miRNAs are generated by Drosha-independent noncanonical pathways (Okamura *et al.* 2007; Ruby *et al.* 2007; Bogerd *et al.* 2010; Valen *et al.* 2011).

Dicer is another RNaseIII endoribonuclease that plays a central role in the processing of certain classes of small RNAs; it is required for the maturation of endogenous siRNAs and miRNAs but not of piRNAs (Bernstein *et al.* 2001; Grishok *et al.* 2001; Vagin *et al.* 2006; Houwing *et al.* 2007). We tested a requirement of Dicer in *DXZ4* small RNA expression by transiently depleting Dicer expression in HEK293T cells (Figure S3B). *DXZ4* small RNA expression was analyzed by determining the expression of two *DXZ4* small RNAs of different size by quantitative RT-PCR: a 36-nucleotide RNA (DXZ4-2259as) and a 22-nucleotide RNA (DXZ4-2355as). Dicer depletion had no significant effect on the abundance of both small RNAs (Figure 2B).

Only RNAs with both the 2′ and 3′ hydroxy termini such as animal siRNAs and miRNAs react with sodium periodate (NaIO_4_) (Elbashir *et al.* 2001; Hutvagner *et al.* 2001). In contrast, piRNAs are modified by 2′-*O*-methylation blocking the NaIO_4_ reaction. NaIO_4_-reacted RNAs migrate faster than unreacted RNA in denaturing gel electrophoresis, generating a signal shift in subsequent Northern hybridizations. This difference provides a direct way to distinguish endogenous siRNAs and miRNAs from piRNAs (Vagin *et al.* 2006; Kirino and Mourelatos 2007). We used this chemical probing to characterize *DXZ4* small RNAs which, regarding their spectrum of sizes, potentially belong to more than one class of small RNA (Figure S2). miR-15a, which was expected to be unmodified at its 3′ end, was sensitive to the treatment and showed a ~2 nucleotide shift. We used two Northern probes to detect *DXZ4* small RNAs in both orientations. Individual RNAs in both the <200 nucleotide population and the chromatin-associated RNA fraction shifted after treatment whereas other RNAs did not. This discriminative behavior of individual small RNAs adds to previous lines of evidence indicating the expression of small RNAs from *DXZ4* with different characteristics.

Inert RNAs are indicative of a block of the NaIO_4_ reaction suggesting a 3′ modification of certain *DXZ4* small RNAs, potentially analogous to as previously documented for piRNAs. Primary piRNAs from Drosophila and mouse testis have a strong preference for uridine at their 5′ position (Girard *et al.* 2006; Aravin *et al.* 2007; Brennecke *et al.* 2007). In this regard, and in reference to their “ping-pong” amplification biogenesis mechanism, secondary piRNAs have an adenine bias at nucleotide position ten (Brennecke *et al.* 2007). In contrast, *DXZ4* small RNAs did not display any nucleotide preference suggesting that they are generated by a ping-pong unrelated mechanism (Figure 2C). Primary piRNAs are generated by mechanisms that are not well understood, particularly those piRNAs generated outside the mammalian germline (Ishizu *et al.* 2012). In certain cell types, such as somatic follicle cells in flies or mouse spermatocytes, piRNAs are only generated by the primary biogenesis pathway (Siomi *et al.* 2011; Yan *et al.* 2011). The absence of multiple copies of the same RNA in the sequencing data suggests that *DXZ4* RNAs are not generated by amplification. However, nucleotide preferences are not prevalent in siRNAs and miRNAs.

In summary, our characterization of *DXZ4* small RNAs suggests that this RNA population likely represents a noncanonical group of RNAs. Although *DXZ4* small RNAs share certain characteristics with known small RNA classes, all together our assays did not lead to a clear assignment to a previously described RNA class. Further characterization will require deep-sequencing approaches to capture the entire population.

### Argonaute proteins associate with *DXZ4* chromatin

**Figure 3:**
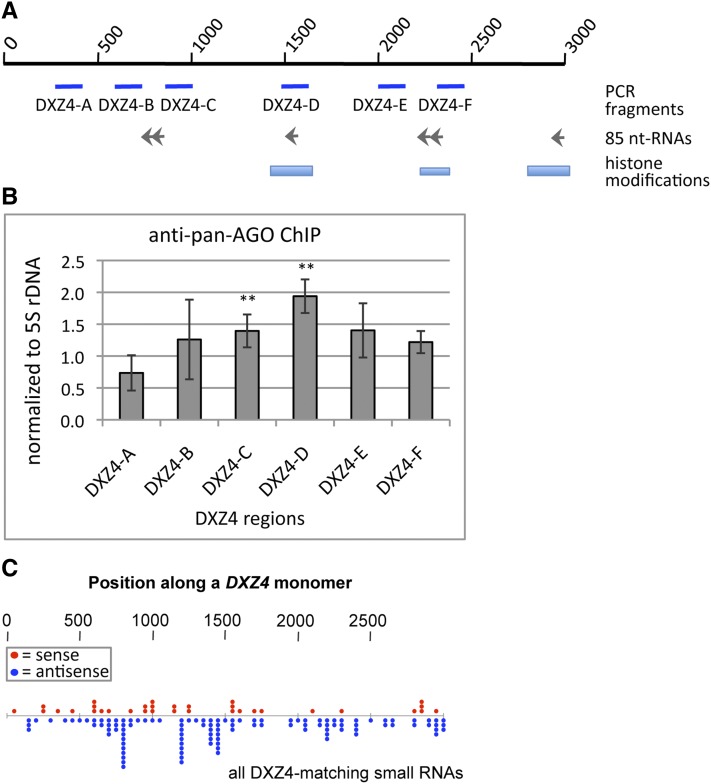
AGO associated with *DXZ4* chromatin. (A) Schematic representation of a *DXZ4* monomer showing fragments amplified to analyze coprecipitated *DXZ4* DNA. Locations of RNAs and histone modifications were adopted from Chadwick (2008). (B) Chromatin immunoprecipitation (ChIP) in HEK293T cells using a pan-AGO antibody. Coprecipitated DNA was quantified by real-time polymerase chain reaction. Relative *DXZ4* enrichment over host matched IgG was normalized to the repetitive 5S rDNA locus (n = 4–5). Double asterisks indicate significant enrichment (*P* < 0.05) calculated by using the *t*-test: DXZ4-C *P*-value = 0.029, DXZ4-D *P*-value = 0.006. (C) Alignment of *DXZ4* matching small RNA reads including alignment to non-*DXZ4* genomic loci with the same or higher stringency. Data obtained from merged high-throughput sequencing data from K562, HEK293, MCF10a, H1, and IMR-90 cell lines (n = 154).

Small RNAs associate with Argonaute proteins to guide them to nascent transcripts in a sequence-specific manner (Grewal and Jia 2007). Based on our finding that several AGO proteins bound a substantial number of *DXZ4* small RNAs (Figure 1E), we asked whether AGO directly associates with *DXZ4*. To address this question, we performed ChIP using a pan-AGO antibody specific for AGO-1 through AGO-4 in HEK293T cells (Nelson *et al.* 2007; Schwartz *et al.* 2008). Coprecipitated DNA was detected in immunoprecipitated chromatin by quantitative PCR and normalized relative to a species-matched IgG control. *DXZ4* was analyzed using a set of PCR fragments dispersed across a *DXZ4* monomer (Figure 3A). We used the *5S rDNA* repeat cluster, which only showed a modest enrichment compared with IgG as a negative control to copy number-match the *DXZ4* array and further normalized *DXZ4* enrichments relative to it. *DXZ4* amplification between base pairs 820 and 1600 of the monomer was significantly enriched relative to the IgG control (Figure 3B).

*DXZ4*-matching RNAs that align to a different genomic locus with the same or a higher stringency irrespective of their origin have an equivalent potential in guiding Argonaute to *DXZ4*. When we compared the ChIP data with the distribution of this comprehensive small RNA population, it became obvious that the fragments with significant ChIP enrichment coincided with sites of mounded small RNA alignment to *DXZ4* (Figure 3C). In summary, these data indicate that one or several members of the AGO subfamily are part of *DXZ4* chromatin.

### Argonaute proteins possibly play a role in DNA methylation at *DXZ4*

The detection of AGO in *DXZ4* chromatin prompted us to investigate a potential functional consequence of AGO association with *DXZ4*. One of the characteristics of *DXZ4* is it’s hypermethylation on the active X chromosome (Giacalone *et al.* 1992; Chadwick 2008). To investigate whether Argonaute proteins are essential in establishing or maintaining *DXZ4* methylation, we determined the effects of AGO-1 depletion on CpG methylation. Furthermore, because it is known that the piRNA pathway is involved in DNA methylation of protein-coding genes and transposons in mouse male germ cells (Aravin *et al.* 2008; Kuramochi-Miyagawa *et al.* 2008; Watanabe *et al.* 2011), we included the PIWI protein PIWIL4, which is expressed in somatic cells (Sugimoto *et al.* 2007). Using RT-PCR, we could confirm PIWIL4 expression in MRC-5, IMR-90, and HEK293T fibroblast lines (Figure S3A). RNAi-mediated knockdown of AGO-1 and PIWIL4 reduced the mRNA of these genes by at least 70% (Figure S3B).

**Figure 4:**
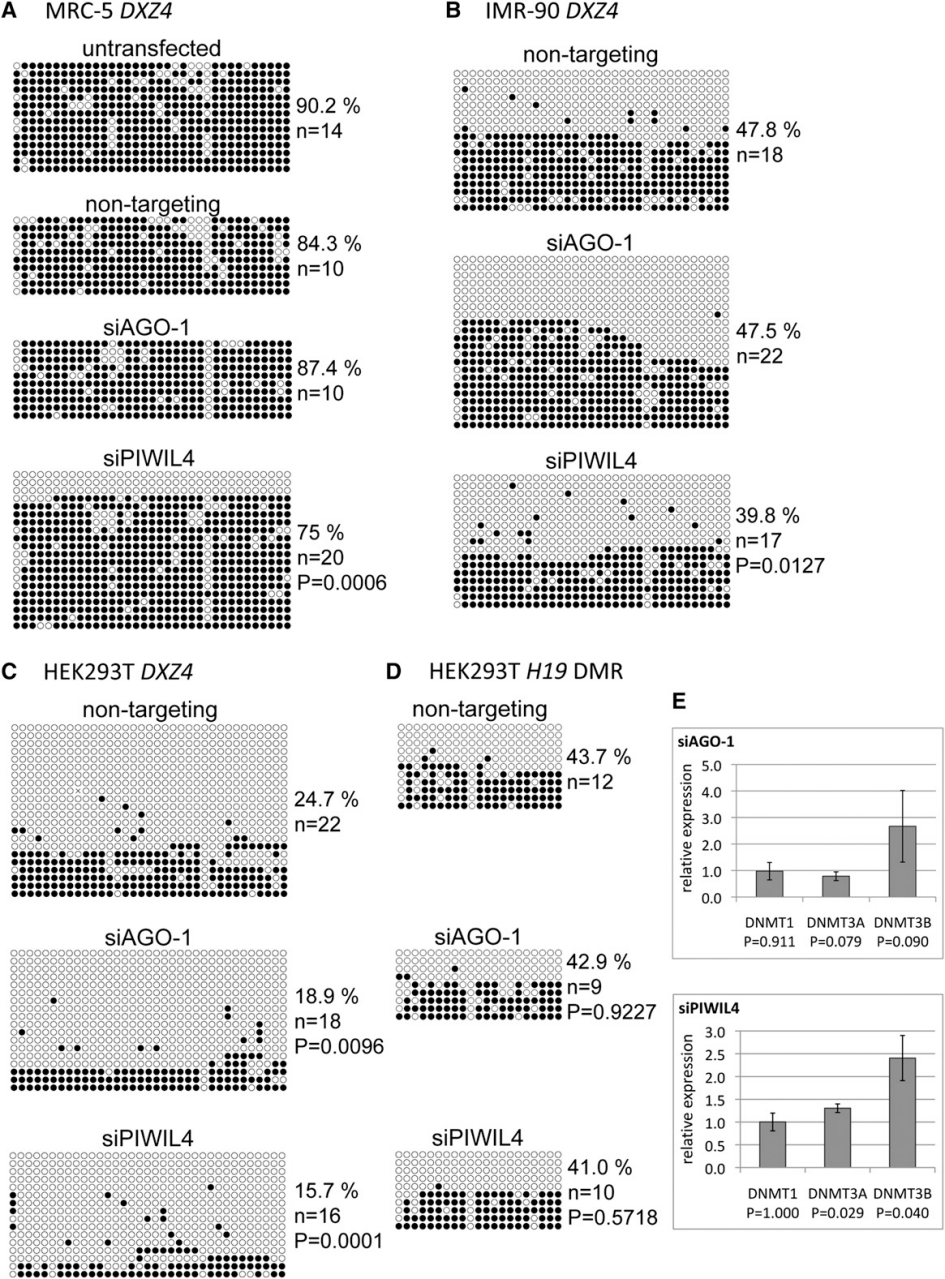
Reduction in CpG methylation at *DXZ4* after Argonaute depletion. (A–C) CpG methylation of a 494-base pair *DXZ4* region was assessed by bisulfite sequencing. Each row of circles represents an individual sequenced clone. Open circles indicate unmethylated and filled circles indicate methylated cytosine residues of a CpG. Percent methylated CpGs and number of sequenced clones are indicated. P-values were calculated by via the Fisher’s exact test for data pairs from cell populations treated with the nontargeting siRNA or with an Argonaute siRNA. (D) Analysis of CpG methylation of the differential methylated region (DMR) of the *H19* locus. (E) mRNA expression levels of DNA methyltransferases in Argonaute-depleted cells relative to nontargeting siRNA-transfected cells determined by quantitative RT-PCR (n = 3–4). P-values were calculated using the *t*-test.

Sequencing of bisulfite-treated genomic DNA isolated from siRNA-transfected cells was used to determine DNA methylation levels. A *DXZ4* fragment was analyzed that covers 35 CpG dinucleotides and contains the internal promoter and the CTCF binding region (Chadwick 2008). This *DXZ4* fragment was nearly completely methylated in the male primary fibroblasts MRC-5, which is analogous to previously published data (Chadwick 2008). Although AGO-1 knockdown did not affect *DXZ4* methylation, depletion of PIWIL4 reduced the number of methylated CpGs statistically significant compared with levels found in mock transfected cells (Figure 4A). We further analyzed *DXZ4* methylation in IMR-90 fibroblasts, which are a female counterpart of MRC-5. As expected, *DXZ4* methylation in these cells was approximately 50% because only *DXZ4* on the active X chromosome is methylated. Argonaute knockdown confirmed the result observed with the MRC-5 cells with a statistically significant reduction of *DXZ4* methylation in PIWIL4 depleted IMR-90 cells (Figure 4B).

We included a second female cell line, HEK293T, in our analysis. In these cells, *DXZ4* DNA methylation in mock transfected HEK293T was at 25% instead of the expected 50%. Because the HEK293T cell line is a derivative of the experimentally transformed female HEK293 line that has an aberrant chromosome content (Graham *et al.* 1977), we determined the active and inactive X chromosome ratio by *in situ* hybridizations. X chromosomes were labeled using a probe of the *DXZ4* monomer in DNA-FISH. Four signals per nucleus were observed in most of the cells, indicating the presence of four X chromosomes (Figure S4). Four X chromosomes also were seen by using a bacterial artificial chromosome clone encompassing the *X inactive-specific transcript (XIST)* locus as a probe (data not shown). To detect the inactive X chromosomes, *DXZ4* DNA-FISH was combined with *XIST* RNA-FISH. The noncoding *XIST* is expressed from and preferentially coats the inactive X, generating a diffuse fluorescence signal marking the X chromosome (Clemson *et al.* 1996). Two *XIST* signals colocalized with *DXZ4* signals, suggesting two of the four X chromosomes were silenced and two should be active (Figure S4). Thus, our data detecting 25% DNA hypermethylation of *DXZ4* in these cells suggest that only one of the two active X chromosomes is expressed from this region. Moreover, it is also possible that *DXZ4* is methylated on only one of the active X chromosomes or the *DXZ4* arrays on both active X chromosomes are incompletely methylated containing methylated and unmethylated monomers accumulating at 25% methylation.

In HEK293T cells, depletion of PIWIL4 again significantly reduced DXZ4 methylation? (Figure 4C). Moreover, AGO-1 depletion resulted in a statistically significant reduction of *DXZ4* CpG methylation as well. In the context of the association of *DXZ4* small RNAs with AGO-1 (Figure 1E), this observation suggests a guiding role of these RNAs for Argonaute in *cis*. The cause of the different AGO-1−dependent outcomes in the different cell types is unknown. A possible explanation could be the presence of compensation mechanisms that are present in primary cells but are lacking in the transformed HEK293T cell line.

To investigate whether Argonaute knockdown induced methylation changes at other loci, we examined CpG methylation at the *H19* locus. We chose *H19* because disruption of the piRNA pathway does not affect *H19* DNA methylation (Watanabe *et al.* 2011). As expected, knockdown of AGO-1 or PIWIL4 did not change DNA methylation globally (Figure 4D).

To consider a potential indirect effect of Argonaute depletion on DNA methylation levels, we determined the effect on the expression of DNA methyltransferases, enzymes that catalyze the addition of methyl groups to DNA. The three known mammalian DNA methyltransferases are expressed in HEK293T cells (Choi *et al.* 2011). Argonaute knockdown did not result in a reduction of *DNMT* levels, indicating that the reduced *DXZ4* methylation was not due to an effect on *DNMT* expression (Figure 4E). Overall, these findings support a role for Argonaute proteins in establishing or maintaining DNA methylation at *DXZ4* in somatic cells. A requirement for PIWIL4 in somatic cell DNA methylation extends previous reports showing a role for components of the piRNA pathway outside of the germline. Although the use of a pan-AGO antibody in our ChIP experiments does not allow us to specify the AGO protein, based on its role in *DXZ4* methylation, we speculate that AGO-1 is likely among the precipitated AGO members.

It has previously been shown that *DXZ4* is expressed from both the active and the inactive X chromosome (Chadwick 2008). Considering the mainly suppressive nature of DNA methylation, a reduction could potentially have a positive effect on *DXZ4* expression. We therefore investigated the effect of AGO-1 and PIWIL4 depletion on *DXZ4* transcription in male MRC-5 cells, which are normally hypermethylated. *DXZ4* expression was analyzed at three regions of the *DXZ4* monomer by quantitative RT-PCR (Figure S5). Only AGO-1 knockdown generated a slight increase in *DXZ4* expression at one of the three regions tested. Taken together, neither AGO-1 nor PIWIL4-mediated reduction in DNA methylation caused a clear change in *DXZ4* transcription output. Because the data presented here indicate that Argonaute and Dicer are not required for *DXZ4* small RNA expression, it is not known by which means these small RNAs are generated. However, it seems plausible that *DXZ4* small RNA expression is generally linked to the transcription of long *DXZ4* RNAs that is controlled by the bidirectional promoter residing within each monomer (Chadwick 2008). How the small RNAs possibly mature from the longer transcripts requires further investigation.

Characterization of the physiological functions of diverse small RNAs has lagged behind their discovery (Valen *et al.* 2011). Here, we describe a potential link between the expression of small RNAs, members of the Argonaute family, and DNA methylation of the macrosatellite *DXZ4* in human somatic cells. Furthermore, we provide additional evidence that the piRNA pathway is active in nongermline cells. In the germline, Piwi functions in maintaining structural integrity of the genome (Mani and Juliano 2013). We speculate that Argonaute/macrosatellite interactions in somatic cells may have similar functions.

## Supplementary Material

Supporting Information
