# Supplementary material for: Small RNA Expression from the Human Macrosatellite DXZ4
Source: G3 (Bethesda). 2014 Aug 21;4(10):1981–9. doi: 10.1534/g3.114.012260 (PMC4199704; doi:10.1534/g3.114.012260)
Supplement: Supporting Information [file supp_4_10_1981__index.html]

Small RNA Expression from the Human Macrosatellite DXZ4 — Supporting Information 

# Small RNA Expression from the Human Macrosatellite DXZ4

## Supporting Information for Pohlers, Calabrese, and Magnuson, 2014

**Files in this Data Supplement:**

- Supporting Information - Figures S1-S5 (PDF, 522 KB)
- Figure S1 - Chromatin association of small RNAs expressed from the *DXZ4* region between nucleotides 2100 and 2400. (PDF, 420 KB)
- Figure S2 - Chemical probing of *DXZ4* small RNA 3' ends. (PDF, 508 KB)
- Figure S3 - Efficiencies of RNAi-mediated depletion of small RNA pathway factors. (PDF, 275 KB)
- Figure S4 - Determination of the ratio of active X chromosomes versus total number of X chromosomes in HEK293T cells. (PDF, 359 KB)
- Figure S5 - *DXZ4* expression after AGO-1 or PIWIL4 siRNA-mediated depletion. (PDF, 483 KB)
