# Supplementary material for: Small RNA Expression from the Human Macrosatellite DXZ4
Source: G3 (Bethesda). 2014 Aug 21;4(10):1981–9. doi: 10.1534/g3.114.012260 (PMC4199704; doi:10.1534/g3.114.012260)
Supplement: Supporting Information [file supp_g3.114.012260_012260SI.pdf]

G3 · Genes | Genomes | Genetics

Small RNA expression from the human macrosatellite DXZ4

Michael Pohlers, J. Mauro Calabrese, and Terry Magnuson

Department of Genetics, the Carolina Center for Genome Sciences, and the Lineberger Comprehensive Cancer Center
University of North Carolina
Chapel Hill, NC 27599, USA

Corresponding author:
Terry Magnuson
Department of Genetics
University of North Carolina at Chapel Hill
5016 Genetic Medicine Bldg., CB # 7264
120 Mason Farm Road
Chapel Hill, NC 27599
USA

Telephone:	+1-9198436475
Fax:	+1-9198436365
Email:	trm4@med.unc.edu

DOI: 10.1534/g3.114.012260

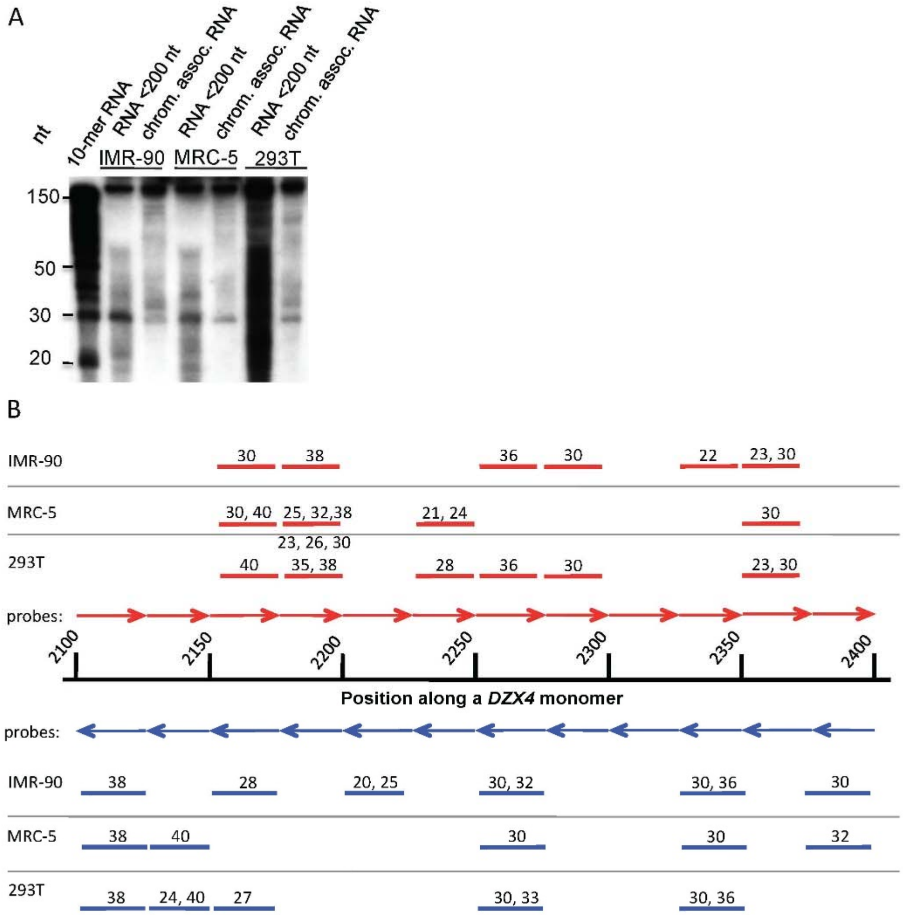

Figure S1 Chromatin association of small RNAs expressed from the *DXZ4* region between nucleotides 2100 and 2400. (A) Example of a small RNA Northern blot detecting the expression of small RNAs from both the total RNA <200 nucleotides and the chromatin-associated RNA from primary fibroblast lines (IMR-90, MRC-5) and from the HEK293T cell line (293T). Relative location of the probe: nucleotides 2326-2350. (B) Summary of detected 19–40 nucleotide long small RNAs from the 300 base pair-region using consecutive 25-nucleotide probes. The numbers above the red and blue bars indicate the size of the small RNAs estimated by comparison to a co-migrated RNA ladder. The arrows indicate location of the probes.

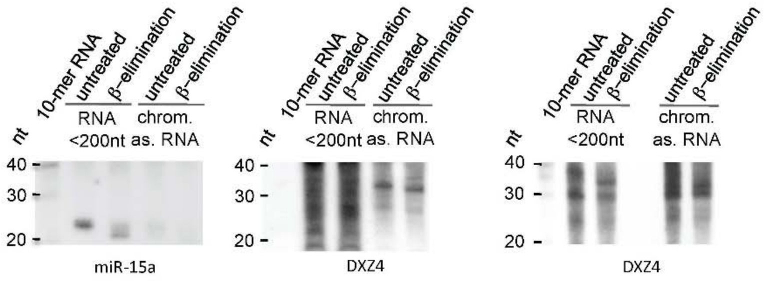

Figure S2 Chemical probing of *DXZ4* small RNA 3' ends. RNA <200 nucleotides and chromatin associated (chrom. as.) RNAs from HEK293T cells were $NaIO_4$-reacted and β-eliminated. *DXZ4* RNAs were detected by Northern hybridization. The 3' unmodified miRNA miR-15a was included as a positive control for chemical treatment.

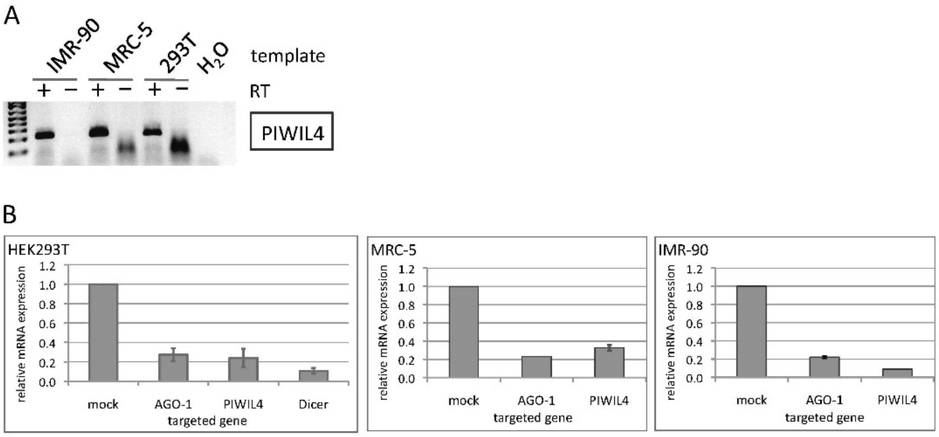

Figure S3 Efficiencies of RNAi-mediated depletion of small RNA pathway factors. (A) Representative example of RT-PCR to detect PIWIL4 expression in human fibroblasts. cDNA syntheses were carried out in the presence (+RT) or absence (–RT) of reverse transcriptase. (B) Quantitative RT-PCR of mRNA expression levels of AGO-1, PIWIL4, and Dicer after transfection of siRNAs targeting the respective gene. Data from n≤6 (HEK293T) and n=1–2 (MRC-5, IMR-90) independent experiments.

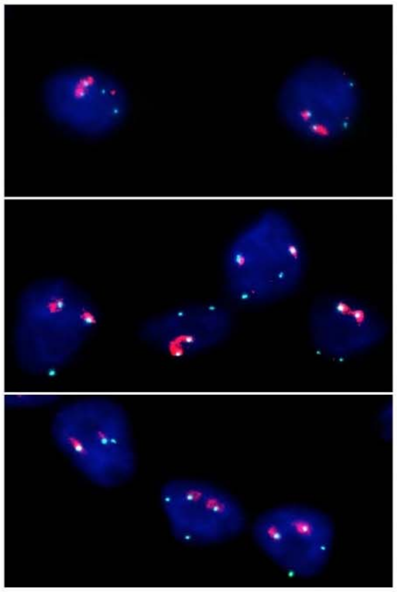

Figure S4 Determination of the ratio of active X chromosomes versus total number of X chromosomes in HEK293T cells. Three examples of X chromosomes labeled by *DXZ4* DNA-FISH (cyan) combined with *XIST* RNA-FISH (red). Nuclear DNA was counterstained with DAPI (blue).

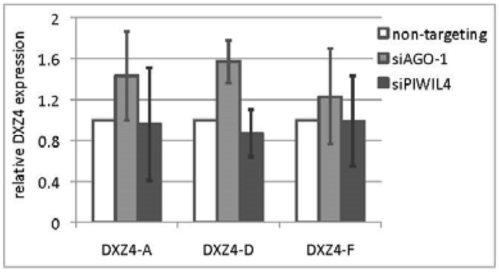

Figure S5 ***DXZ4*** **expression after AGO-1 or PIWIL4 siRNA-mediated depletion.** Expression in MRC-5 fibroblasts was determined by detecting *DXZ4* RNA levels from three regions (see Figure 3A) by quantitative RT-PCR [n=2]. Shown are ratios relative to non-targeting siRNA transfections.
