# Supplementary material for: Small RNA Expression from the Human Macrosatellite DXZ4
Source: G3 (Bethesda). 2014 Aug 21;4(10):1981–9. doi: 10.1534/g3.114.012260 (PMC4199704; doi:10.1534/g3.114.012260)
Supplement: Supporting Information [file supp_g3.114.012260_FigureS1.pdf]

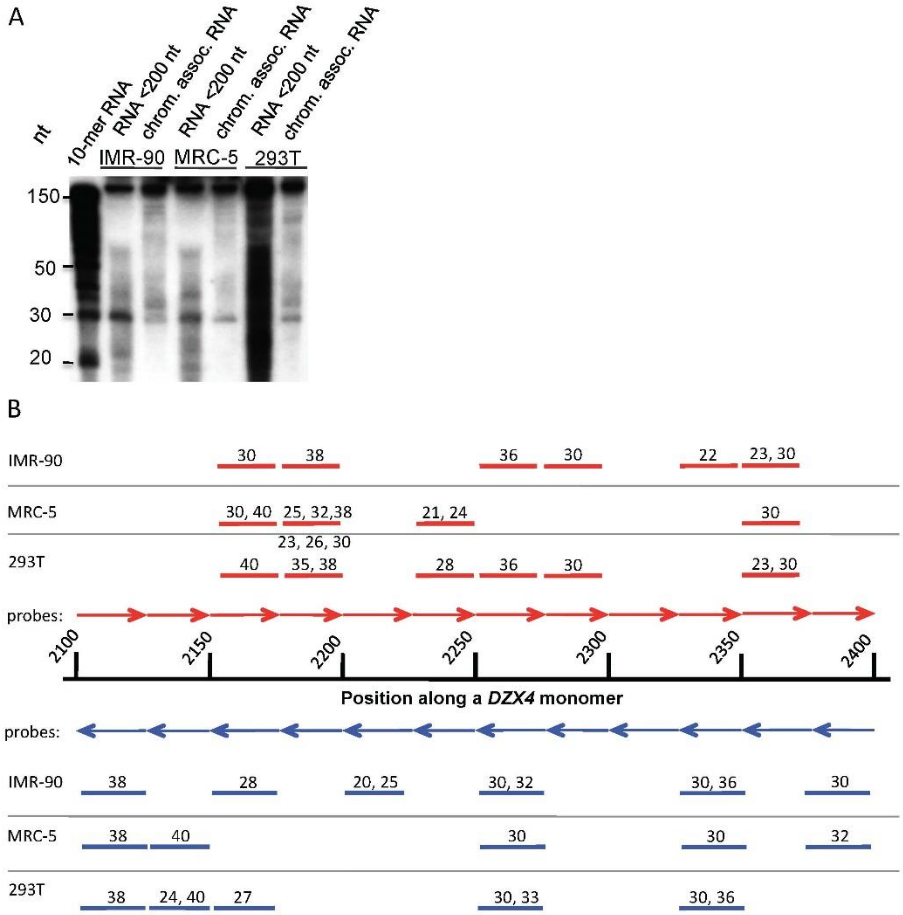

Figure S1 Chromatin association of small RNAs expressed from the *DXZ4* region between nucleotides 2100 and 2400. (A) Example of a small RNA Northern blot detecting the expression of small RNAs from both the total RNA <200 nucleotides and the chromatin-associated RNA from primary fibroblast lines (IMR-90, MRC-5) and from the HEK293T cell line (293T). Relative location of the probe: nucleotides 2326-2350. (B) Summary of detected 19–40 nucleotide long small RNAs from the 300 base pair-region using consecutive 25-nucleotide probes. The numbers above the red and blue bars indicate the size of the small RNAs estimated by comparison to a co-migrated RNA ladder. The arrows indicate location of the probes.
