# Supplementary material for: Small RNA Expression from the Human Macrosatellite DXZ4
Source: G3 (Bethesda). 2014 Aug 21;4(10):1981–9. doi: 10.1534/g3.114.012260 (PMC4199704; doi:10.1534/g3.114.012260)
Supplement: Supporting Information [file supp_g3.114.012260_FigureS2.pdf]

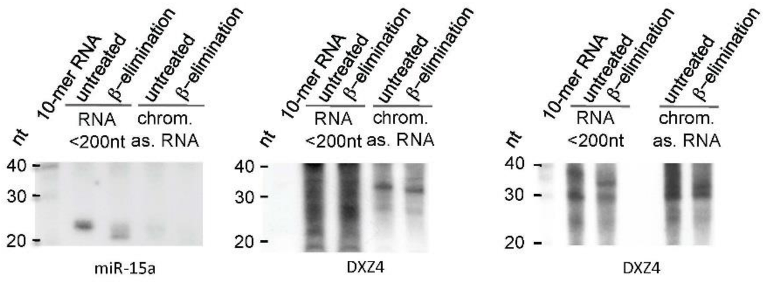

Figure S2 Chemical probing of *DXZ4* small RNA 3' ends. RNA <200 nucleotides and chromatin associated (chrom. as.) RNAs from HEK293T cells were $NaIO_4$-reacted and β-eliminated. *DXZ4* RNAs were detected by Northern hybridization. The 3' unmodified miRNA miR-15a was included as a positive control for chemical treatment.
