# Supplementary material for: Small RNA Expression from the Human Macrosatellite DXZ4
Source: G3 (Bethesda). 2014 Aug 21;4(10):1981–9. doi: 10.1534/g3.114.012260 (PMC4199704; doi:10.1534/g3.114.012260)
Supplement: Supporting Information [file supp_g3.114.012260_FigureS3.pdf]

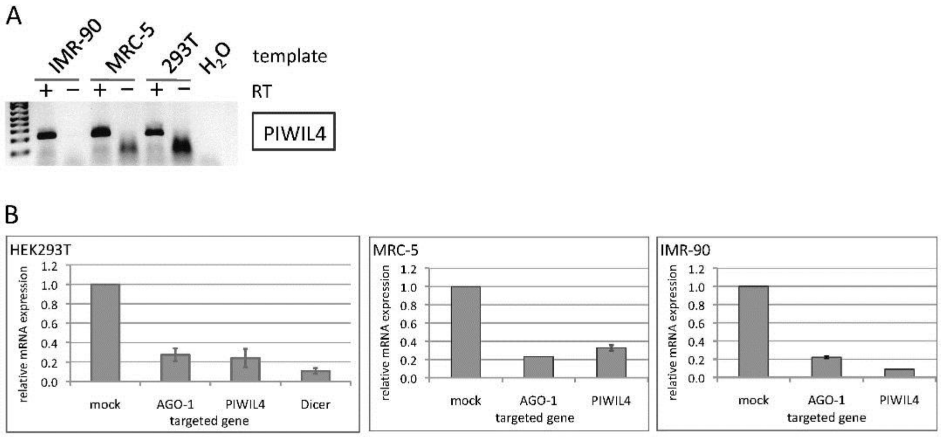

Figure S3 Efficiencies of RNAi-mediated depletion of small RNA pathway factors. (A) Representative example of RT-PCR to detect PIWIL4 expression in human fibroblasts. cDNA syntheses were carried out in the presence (+RT) or absence (–RT) of reverse transcriptase. (B) Quantitative RT-PCR of mRNA expression levels of AGO-1, PIWIL4, and Dicer after transfection of siRNAs targeting the respective gene. Data from n≤6 (HEK293T) and n=1–2 (MRC-5, IMR-90) independent experiments.
