# Supplementary material for: Small RNA Expression from the Human Macrosatellite DXZ4
Source: G3 (Bethesda). 2014 Aug 21;4(10):1981–9. doi: 10.1534/g3.114.012260 (PMC4199704; doi:10.1534/g3.114.012260)
Supplement: Supporting Information [file supp_g3.114.012260_FigureS4.pdf]

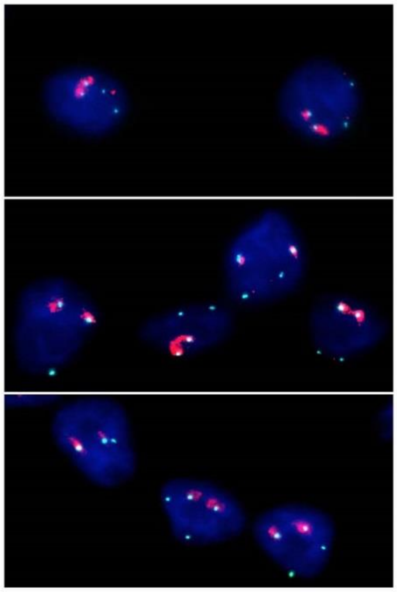

Figure S4 Determination of the ratio of active X chromosomes versus total number of X chromosomes in HEK293T cells. Three examples of X chromosomes labeled by *DXZ4* DNA-FISH (cyan) combined with *XIST* RNA-FISH (red). Nuclear DNA was counterstained with DAPI (blue).
