# Supplementary material for: Small RNA Expression from the Human Macrosatellite DXZ4
Source: G3 (Bethesda). 2014 Aug 21;4(10):1981–9. doi: 10.1534/g3.114.012260 (PMC4199704; doi:10.1534/g3.114.012260)
Supplement: Supporting Information [file supp_g3.114.012260_FigureS5.pdf]

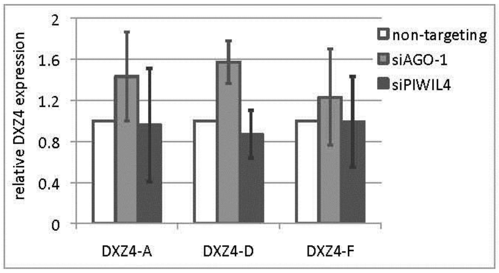

Figure S5 ***DXZ4*** **expression after AGO-1 or PIWIL4 siRNA-mediated depletion.** Expression in MRC-5 fibroblasts was determined by detecting *DXZ4* RNA levels from three regions (see Figure 3A) by quantitative RT-PCR [n=2]. Shown are ratios relative to non-targeting siRNA transfections.
